# A New Method of Vaccination

**Published:** 1900-02-24

**Authors:** 


### A NEW METHOD OF VACCINATION.

A new dodge in vaccinating comes to us from New York, one, however, which we think will not commend itself to practical practitioners. The idea is to denude the skin preparatory to vaccination by the use of caustic potash, instead of by the ordinary method of scarification. According to Dr. Fielder, who described the experiments to one of the New York societies, the use of the caustic is recommended in the case of nervous children, since it obviates the use of any instrument and the drawing of any blood. Moreover, it was stated that by this method the pain of vaccination is obviated. Dr. Fielder's experiments seemed to justify these claims, but that is not everything. The pain of vaccination is not in the operation, but afterwards; and when we find that more than 30 per cent. of the cases failed to "take," and that this seemed due to a parchment-like condition of the skin produced by the potash, we do not feel much drawn to any such manœuvre. If only people can be brought to the point and be got to consent to vaccination, it is throwing away a good opportunity to dabble with potash and run the risk of 30 per cent. of failures, instead of vaccinating them properly at once.

